# Assessment of fluoride bio-accessibility in early childhood diets

**DOI:** 10.3389/froh.2025.1526262

**Published:** 2025-02-06

**Authors:** Jelena Kronic, Ralph M. Duckworth, Claudio Angione, Steven M. Levy, Fatemeh Vida Zohoori

**Affiliations:** ^1^ School of Health and Life Sciences, Teesside University, Middlesbrough, United Kingdom; ^2^ School of Computing, Engineering and Digital Technologies, Teesside University, Middlesbrough, United Kingdom; ^3^ College of Dentistry and Dental Clinics, University of Iowa, Iowa City, IA, United States

**Keywords:** fluoride, infant, child, bio-accessibility, diet, Iowa fluoride study

## Abstract

**Introduction:**

Currently available dietary recommendations for fluoride provided as “adequate intake” (AI) and “tolerable upper intake level” (UL) assume 100% fluoride availability for utilization by the body, which is often not the case. To prevent the development of dental fluorosis, AI and UL values must include fluoride bioavailability. However, the lack of data on fluoride bioavailability/bio-accessibility has hindered progress so far. This study aimed to measure fluoride bio-accessibility of the dietary sources commonly consumed by children below four years of age.

**Methods:**

A total of 103 food and meal samples were purchased, prepared, and analyzed for fluoride content, subjected to a standardized static *in vitro* digestion procedure and subsequent determination of fluoride concentration of resultant mixtures necessary for the final fluoride bio-accessibility calculation. Fluoride concentrations before and after *in vitro* digestion were determined directly using a fluoride-ion-selective electrode after addition of TISAB III, or indirectly by an acid diffusion method. Additionally, fluoride concentration of milk samples was determined using a combination of microwave-assisted acid digestion and the direct method of fluoride analysis.

**Results:**

Mean (SD) fluoride bio-accessibility for individual food samples was 44.7% (37.5%). The mean (SD) fluoride bio-accessibilities for meals created with juice, carbonated drinks, tap water, and milk were 79.0% (21.9%), 64.3% (20.7%), 40.2% (20.9%), and 71.5% (17.1%), respectively. For the rest of the meals with no common mixing agent, the mean (SD) fluoride bio-accessibility was 50.8% (55.9%).

**Conclusion:**

The majority of dietary sources analyzed in this project resulted in fluoride bio-accessibilities below 100%, indicating incomplete utilization of consumed fluoride. As the first study of its kind, these findings represent a critical initial step for future research and provide valuable insights to inform policymakers and health authorities in revising fluoride intake guidelines.

## 1 Introduction

Although fluoride (F) is important for the prevention of dental caries (1, 2), excessive systemic fluoride intake in infancy and early childhood may result in the development of dental fluorosis (3). While different timeframes have been suggested in the literature (4–7), the first three years of life are generally considered to be the most critical for the development of dental fluorosis on the aesthetically important permanent maxillary central incisors (5). To minimize the risk of dental fluorosis development, it is crucial to ensure fluoride intake remains within recommended guidelines. However, an increase in the occurrence of dental fluorosis has been noted in many industrialized countries (8–11) as a potential consequence of increased fluoride exposure through dietary as well as non-dietary sources along with increased globalization and movement of dietary products from fluoridated areas of production to fluoridated and non-fluoridated areas of consumption (12).

To monitor fluoride intake, a range of 0.05 and 0.07 mg of fluoride per kilogram of body weight per day (mg F/kg bw/day), commonly referred to as the “optimal” fluoride intake, has been in use since the 1940s (13, 14). Additional terminologies like “adequate intake” (AI) of 0.05 mg F/kg bw/day (15) and “tolerable upper intake level” (UL) of 0.1 mg F/kg bw/day for children between 1 and 8 years of age (16) were developed by various international organizations (17–19) to describe fluoride intake levels that reduce the probability of dental caries while simultaneously presenting minimal risk of dental fluorosis. However, the “optimal” fluoride intake was empirically determined in 1943 based on the average fluoride intake from diets commonly consumed by children between 1 and 12 years of age (14, 20). Studies looking to provide concrete evidence on the “optimal” fluoride intake failed to do so, prompting the suggestion that the term “optimal” fluoride intake should be removed from common usage (20, 21). Furthermore, recommended intake levels were created based on the assumption that 100% of consumed fluoride is available for utilization by the body, which is often not the case (22). Factors like diet and age can affect overall fluoride ingestion, absorption, and retention (23, 24). Hence dental fluorosis may rather be a consequence of fluoride bioavailability and it is important to measure the amount of absorbed and body-retained fluoride rather than only the absolute fluoride intake (25). The “San Francisco Fluoride Symposium - 2017” highlighted the significance of fluoride metabolism research, specifically focusing on overall fluoride bioavailability, as such research could lead to evidence-based information needed for improvement of presently available AI and UL values for fluoride (26). Furthermore, insights into fluoride bioavailability could help with Global Target 2.2 of the World Health Organization Global Health Action Plan, which aims for a minimum of 50% of countries to adopt national guidance ensuring optimal fluoride delivery to the population by 2030 (27).

However, current knowledge of fluoride bioavailability is limited. Bioavailability of any nutrient is defined as the amount of nutrient that is released in the gastrointestinal tract, enters the systemic circulation, and is used by the body for different biological functions (28–30). Although absolute fluoride bioavailability was detected in earlier studies after sodium fluoride (NaF) ingestion by healthy adults upon fasting (31, 32), the presence of food reduced fluoride bioavailability by almost 50% (32, 33). Villa (34) found 58% fluoride bioavailability from tea consumed on a fasting stomach and 32% when consumed with solids, while more complex fluoride sources like fish or bone-meal tablets revealed fluoride bio-availabilities of only 30% and 5%, respectively (34, 35). Similarly, fluoride bioavailability of commonly consumed diets from different parts of India ranged from 1.6% to 31.7% for north Indian vegetarian and south Indian non-vegetarian dishes, respectively (36).

Despite numerous studies on fluoride bioavailability, researchers mainly focused on simple fluoride sources like NaF, failing to examine its bioavailability from more complex dietary sources of low fluoride concentration. The bioavailability of any nutrient is determined using *in vivo* methods. Although most accurate, *in vivo* methods are often lengthy, costly, and ethically questionable (37), especially when participants are young children and the study involves invasive procedures (i.e., blood donation) (38). A suitable alternative was found in “bio-accessibility” - the amount of nutrient that is released from food in the gastrointestinal tract during digestive processes and is available for absorption (30). Bio-accessibility is measured using *in vitro* methods that simulate different stages of gastrointestinal digestion. *In vitro* digestion models range from simple static systems to multi-compartmental dynamic systems (39). Although dynamic models simulate the human gastrointestinal tract more accurately, they mainly focus only on one specific part of digestion. Furthermore, available dynamic models are in different developmental stages requiring further validation. On the other hand, although simple, standardized static *in vitro* digestion models simulate the three phases of digestion (oral, gastric, and intestinal) and allow for inter-laboratory comparison. This type of experiment requires commonly available laboratory equipment; it is cost-effective, quick, and without ethical constraints (39). Fluoride bio-accessibility experiments are rare. One study conducted on tea infusions showed 91.4% of fluoride bio-accessible after the gastric stage and 92.1% after the gastrointestinal stage. The addition of milk reduced fluoride bio-accessibility by approximately 15% (40).

Considering the importance of fluoride for dental health and complexities related to its bioavailability, this study aims to remove current gaps in knowledge by measuring fluoride bio-accessibility in commonly consumed dietary sources by children during their first three years of life, increasing the understanding of its impact on oral health and promoting improvement of currently available dietary recommendations.

This paper is part of a larger study (41, 42) that aimed to examine the association between fluoride bio-accessibility and dental fluorosis on permanent teeth. This study utilized information gathered through the Iowa Fluoride Study (IFS) – a comprehensive, longitudinal study that aimed to assess the relationship between long-term fluoride exposure from dietary and non-dietary sources and different health outcomes (21, 43–47). The IFS involved the recruitment of families with newborns from eight Iowa hospitals between 1992 and 1995 (5, 21, 43, 44, 48). Assessment of fluoride exposure involved data collection using questionnaires that were sent to participants every few months starting from when a child was 1.5 months of age (43) alongside 3-day food diaries that involved data collection over 2 weekdays/1 weekend day at 1.5, 3, 6, 9, and 12 months of age, continuing every 4–6 months until they were 8.5 years old (49, 50).

## 2 Materials and methods

### 2.1 Selection of food/meals

Copies of the 3-day food diaries gathered in the IFS were obtained from the University of Iowa and analyzed using RStudio 4.2.2 and MS Excel 365 to identify the most commonly consumed dietary sources by children who participated. As the first three years of life were earlier highlighted as the most critical for dental fluorosis development on permanent maxillary central incisors (5), this period became the main focus of the present study. Therefore, the 3-day food diaries were analyzed at six time points: 1.5, 3, 6, 12, 24, and 36 months of age. The analysis involved 91 participants for whom 3-day food diaries were available at all time points of interest (41). A list of dietary sources most commonly consumed by the children was compiled (see Supplementary Tables S1, S2).

### 2.2 Sample preparation

For the 34 individual foodstuffs identified, five sub-samples were purchased online or at local supermarkets in Middlesbrough, UK. Analyzed samples were aggregate samples, comprising equal amounts of each sub-sample purchased. Furthermore, two tap water samples were obtained in Middlesbrough and Newcastle representing non-fluoridated (0.083 µg/ml) and fluoridated water (0.983 µg/ml), respectively. Depending on the sample, individual foodstuffs were analyzed as they were or mixed with water according to the manufacturer's instructions. Samples were prepared using double deionized water, non-fluoridated, and fluoridated tap water. All samples were purchased between October 2021 and March 2022.

Additionally, the food diaries identified 67 meals consumed by the 91 participants, which were created by mixing two or more previously identified individual food samples. Mixing was determined according to the recommended calorie intake for children (51) and the recommended daily intake of each product marked on the package to ensure the most accurate representation of meals consumed by children. The quantities of each individual food sample used for the creation of meals were expressed as ratios calculated using Equation 1

(1) $$R=\frac{I\;}{T\;}$$

where

R = ratio of individual food sample

I = weight of individual food sample (g)

T = total weight of meal (g)

### 2.3 Measurement of fluoride concentration

#### 2.3.1 Laboratory measured fluoride concentration

The fluoride concentration of tap water samples was determined directly using a fluoride ion-selective electrode (Orion Research, model 96–09) upon adding TISAB III. The rest of the food/meal samples were analyzed using the HMDS-facilitated diffusion method (52, 53). All samples were analyzed in triplicate. The fluoride concentration (µg/g) of each sample was then calculated as the average of each triplicate. Additionally, semi-skimmed milk (UK equivalent of 2% milk) and whole milk samples were analyzed using a microwave-assisted acid digestion procedure, which served as a pre-treatment to the direct method of fluoride analysis to allow for the complete extraction of fluoride from food where fluoride might exist bound to cations that prevent detection of fluoride ions (54). In short, 1 g of each sample was weighed in a Teflon vessel, covered with 8 ml of 7 mol/L HNO_3_, and subjected to microwave digestion for 15 min at 180°C. Once cooled down, samples were neutralized with NaOH (8 mol/L and 1.8 mol/L stock solutions) before direct fluoride analysis.

#### 2.3.2 Calculated fluoride concentration of meals

A hypothesis indicating that the fluoride concentration of a meal would match the sum of the fluoride concentrations of individual food samples used for its preparation (expected value) was developed. Expected values were calculated using Equation 2

(2) $$E\;=\sum_{i=1}^{n}R_{i}*F_{i}$$

where

E = expected fluoride content of meal (µg/g),

R_i_ = ratio of the *i*-th food sample used to create a meal,

F_i_ = fluoride concentration of the *i*-th food sample used to create a meal (µg/g),

*n* = number of food samples used to create a meal.

#### 2.3.3 *In vitro* digestion and calculation of fluoride bio-accessibility

One hundred and three food/meal samples were duplicated and subjected to a standardized static *in vitro* digestion procedure that mimics oral, gastric, and small intestinal phases of human digestion (55, 56). Before digestion, chewing of solid food/meal samples was mimicked using a household blender (IMURZ, TC-18), converting food into small enough particles that would be safe for swallowing. Five grams of each sample were then placed into 50 ml centrifuge tubes. The oral phase involved the addition of human salivary *α*-amylase (Sigma-Aldrich, A1031) and simulated salivary fluid (SSF) followed by a short incubation of approximately 2 min accompanied by manual stirring. The gastric phase of the digestion began with the addition of pepsin (Sigma-Aldrich, P7012) and pH adjustment to∼3.0. Incubation was performed at 37°C for 2 h at 150 rpm in an orbital shaker/incubator (Sartorius Stedim Biotech GmbH, CERTOMAT® BS-1). The final phase of the digestion included the addition of bile (Sigma-Aldrich, B3883) and pancreatin (Sigma-Aldrich, P7545), adjustment of the pH value to∼7.0, and incubation at 37°C for 2 h at 150 rpm in the orbital shaker/incubator. Once completed, digestion was stopped by placing the centrifugation tubes in ice, which stopped enzymatic activity. Samples were centrifuged (Eppendorf, 5810 R) at 3,900 rpm for 20 min and the resultant supernatants were stored frozen until further analysis. Fluoride concentrations of supernatants were determined in triplicate using the HMDS-facilitated diffusion method (52, 53).

Alongside food/meal samples, NaF standards of different concentrations (0.1, 1.0, and 10.0 µg/ml) were subjected to the same *in vitro* digestion procedure described above, as earlier bio-accessibility studies indicated different fluoride bio-accessibility values detected between gastric and whole gastrointestinal digestion (40, 57). To examine this further, standards were analyzed using (a) *in vitro* digestion consisting only of the oral and gastric phases of the digestion, and (b) the whole gastrointestinal digestion process.

Final fluoride bio-accessibility was calculated using the following equation:

(3) $$\mathrm{Bio}\text{-}\mathrm{accessibility}\;(\text{\%})=\frac{F_{2}\;}{F_{1}\;}$$

where

F_1_ = fluoride content of the sample determined before *in vitro* digestion (μg/g), and

F_2_ = fluoride content determined after *in vitro* digestion (bio-accessible fraction) (μg/g).

#### 2.3.4 Validity and reliability of the analytical method

To check the validity of the analytical method, a known concentration of fluoride standard (NaF) was added to approximately 10% (*n* = 11) of the samples. These samples were then analyzed in triplicate to measure the recovery of added fluoride. To check reliability, fluoride measurement of approximately 20% of samples (*n* = 24) was repeated. Samples were analyzed in triplicate and double-distilled, deionized water was used as a blank.

#### 2.3.5 Statistical analysis

To compare laboratory-measured and expected fluoride concentrations of meals, a Bland-Altman plot was created (58) using RStudio 4.2.2. This procedure is a standard approach that allows comparison between two methods of measurement, usually a new method and an already established one, and it helps with the decision of whether the new method is acceptable. The analysis involved plotting the average of two methods against the difference between them, enabling a visual assessment of their agreement. The methods are considered to show good agreement if the points on the scatterplot lie close to the middle line.

## 3 Results

### 3.1 Validity and reliability of measurements

The mean (SD) recovery of fluoride added to food/meal samples was 103.4 (7.6)%. The overall recovery ranged from 92.0% to 117.3% detected for fruit-based baby food and peanut butter, respectively. Re-analysis of 24 samples resulted in a mean (SD) difference in fluoride concentration of 0.002 (0.012) µg/g with all the results being within 0.03 µg F/g.

### 3.2 Most commonly consumed food/meals

Analysis of 1,000 individual food diaries during infancy (1.5–6 months of age) and early childhood (12–36 months of age) for the frequency of consumption revealed a final list of 36 individual food items and 67 meals (Supplementary Tables S1, S2, respectively) as the most commonly consumed food/meal samples by children during the first three years of their life (41).

### 3.3 Fluoride concentration of food/meals

#### 3.3.1 Individual food items

Details on fluoride concentration (µg/g) of individual food samples are found in Supplementary Table S1. The overall mean (SD) fluoride concentration of the 36 individual food items was 0.518 (1.811) µg/g. Ten food samples analyzed as representatives of infancy resulted in a mean (SD) fluoride concentration of 0.136 (0.095) µg/g ranging from 0.011 µg/g to 0.249 µg/g for powdered infant formula and vegetable-based baby foods, respectively. Food samples regularly consumed during early childhood (*n* = 26) resulted in a mean (SD) fluoride concentration of 0.665 (2.123) µg/g ranging from 0.023 µg/g to 10.730 µg/g for fruit and canned fish in tomato sauce, respectively.

Food samples available in the UK and US markets were also analyzed in this study (Table 1) indicating mean (SD) fluoride concentrations of 0.097 (0.093) µg/g and 0.195 (0.089) µg/g for the UK and US market, respectively.

**Table 1:** Fluoride concentrations (µg/g) of foods available in the UK and US markets.

UK/US food samples	Fluoride concentration (µg/g) by origin	
	UK	US
Digestive cookies/Graham	0.036	0.217
Baby food fruit	0.049	0.199
Baby food vegetable	0.249	0.228
Baby food cereals	0.173	0.164
Formula powder	0.011	0.047
Crackers/Saltine	0.063	0.317
Mean ± standard deviation	0.097 ± 0.093	0.195 ± 0.089

#### 3.3.2 Meal items

**Figure 1:**
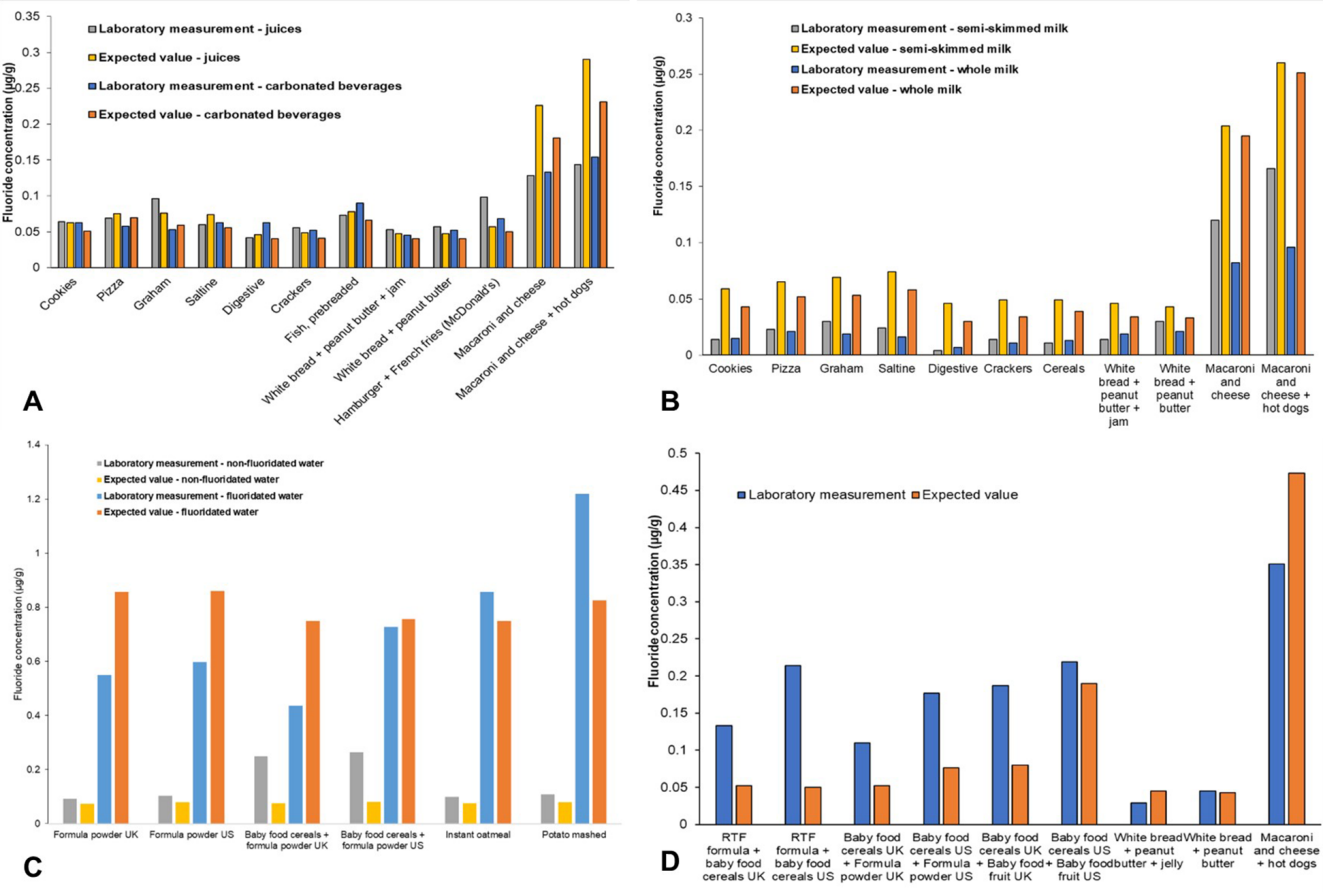
Laboratory-measured and expected (calculated) fluoride concentrations (µg/g) of meal samples when consumed with **(A)** juices or carbonated beverages; **(B)** whole or semi-skimmed (2%) milk; **(C)** fluoridated or non-fluoridated water; and **(D)** other meal samples without a common preparation method.

Sixty-seven analyzed meal samples were grouped according to preparation method. The overall mean (SD) laboratory-measured fluoride concentration of meals was 0.140 (0.213) µg/g while the overall mean expected value (SD) equaled 0.152 (0.219) µg/g. When groups were examined, the highest laboratory-measured mean (SD) fluoride concentration was detected for meals created with water: 0.442 (0.360) µg/g. The corresponding expected fluoride concentration was almost identical: 0.439 (0.379) µg/g. A graphical comparison between laboratory-measured and expected fluoride concentrations for each group is found in Figure 1. Further details are available in Supplementary Table S2.

**Figure 2:**
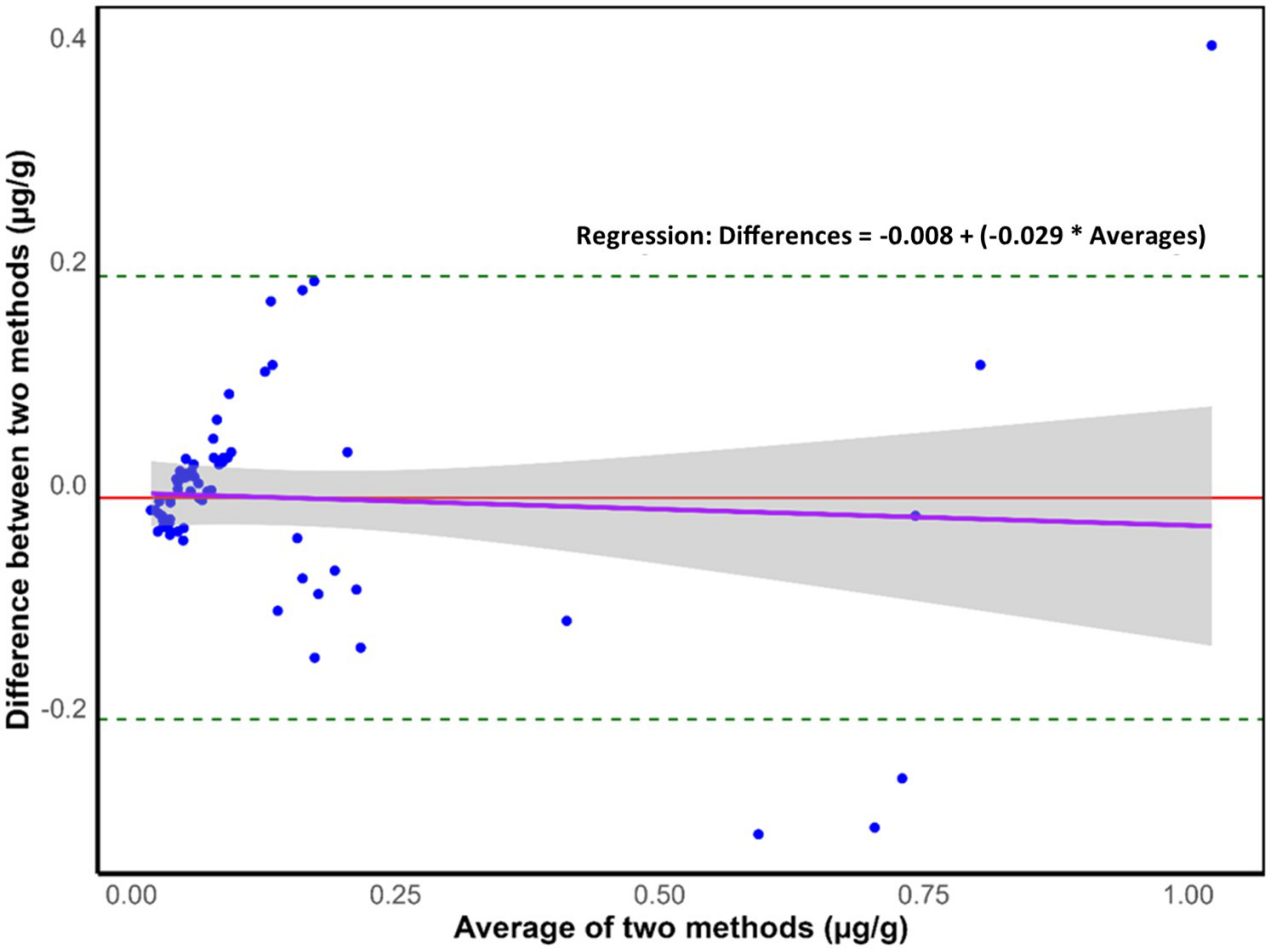
Bland–Altman plot where dashed lines represent the 95% upper and lower limits of agreement (loAs), the middle red line represents the mean difference, the purple line represents the regression line (*n* = 67), and the grey shaded area around the regression line indicates the 95% confidence interval for the regression line.

Figure 2 shows a Bland-Altman plot, where the average of the two methods is shown on the *x*-axis while the difference between the two is plotted on the *y*-axis of the scatterplot. The analysis was conducted using “laboratory-measured” and “expected” fluoride concentrations (µg/g) for 67 meal samples. The average difference (−0.012 µg F/g) is represented by the middle line on the scatterplot while the upper and lower lines represent the limits of agreement (LoA). The upper and lower LoAs (0.186 and −0.210 µg F/g, respectively) were calculated as average difference ± 1.96*standard deviation of the difference. The plot shows a small mean difference between the measured and expected values and narrow LoAs, indicating a good agreement between the methods.

### 3.4 Fluoride bio-accessibility

#### 3.4.1 NaF standards

When small intestinal digestion was excluded from the procedure, the measured fluoride bio-accessibilities were 64.7, 77.6, and 100.1% for the 0.1, 1.0, and 10.0 µg/ml fluoride standards, respectively. The corresponding whole gastrointestinal digestion results were: 56.9, 60.0, and 66.2%, respectively.

#### 3.4.2 Individual food items

Details on fluoride bio-accessibility (%) of individual food samples are found in Supplementary Table S1. The overall mean (SD) fluoride bio-accessibility of the 36 individual food items was 44.7% (37.5%). Ten food samples analyzed as representatives of infancy resulted in a mean (SD) fluoride bio-accessibility of 24.2% (11.8%) ranging from 0.1% to 46.7% for powdered infant formula available on the UK market and fruit-based baby food available on the US market, respectively. Food samples regularly consumed during early childhood (*n* = 26) resulted in a mean (SD) fluoride bio-accessibility of 52.6% (41.1%) ranging from 1.0% to 152.3% for canned fish in tomato sauce and jam, respectively.

#### 3.4.3 Meal items

**Figure 3:**
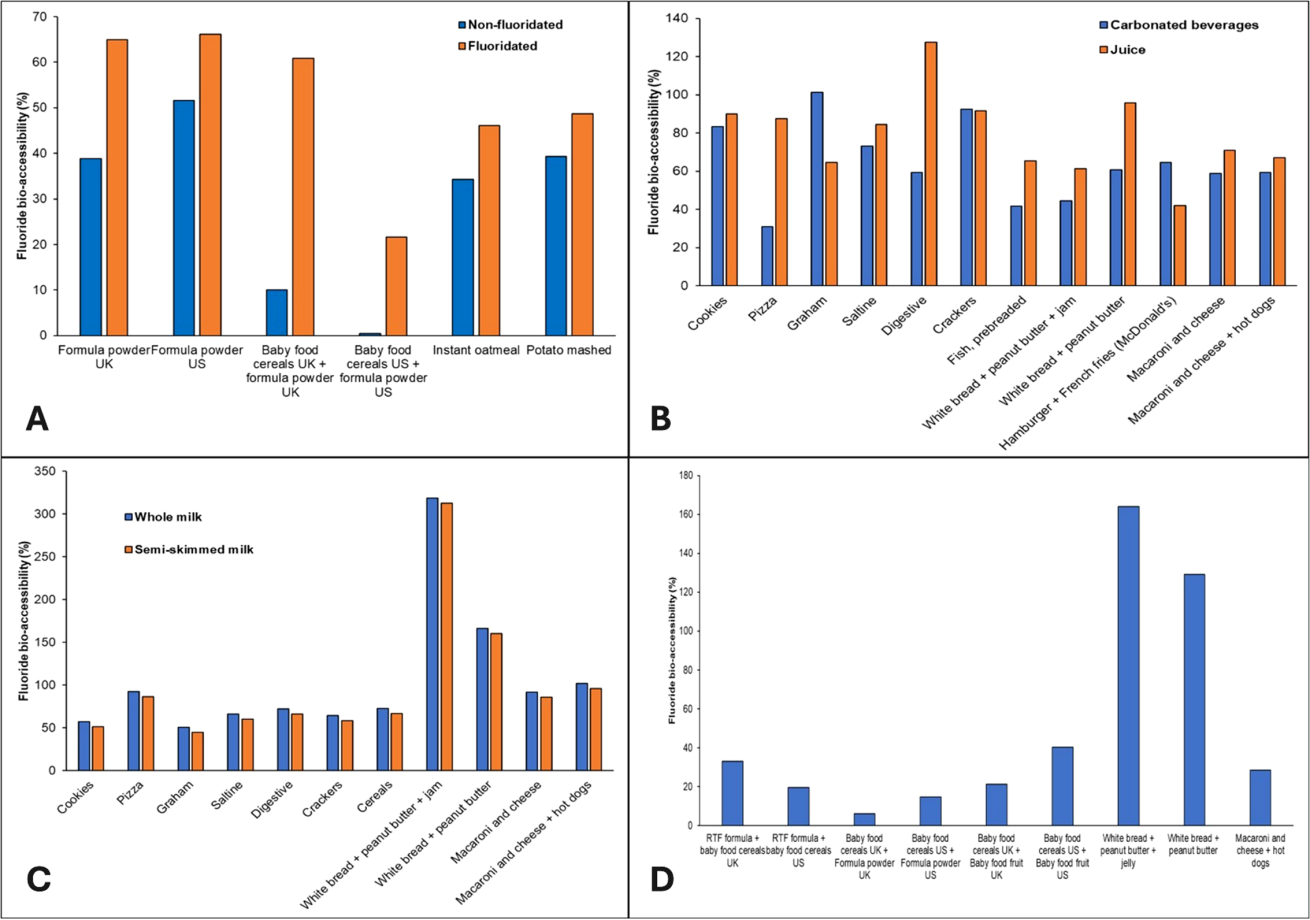
Fluoride bio-accessibility (%) of meal samples when consumed with **(A)** fluoridated or non-fluoridated water; **(B)** carbonated beverages or juice; **(C)** whole or semi-skimmed (2%) milk; and **(D)** other meal samples without a common preparation method.

The mean (SD) fluoride bio-accessibilities for meals when consumed with juice, carbonated drinks, tap water, and milk were 79% (21.9%), 64.3% (20.7%), 40.2% (20.9%), and 102.0% (75.8%), respectively. For the rest of the meals, the average fluoride bio-accessibility was 50.8% (55.9%) (Supplementary Table S4). Figure 3A shows higher fluoride bio-accessibilities recorded for those meals prepared with fluoridated tap water compared with those prepared with non-fluoridated water. Furthermore, all meals shown in Figure 3A resulted in fluoride bio-accessibilities below 100%. Figure 3B shows higher fluoride bio-accessibilities for a majority of meal samples created with juice when compared to those created with carbonated beverages with a notable outlier resulting in 127% bio-accessibility when digestive cookies are consumed with juice. High fluoride bio-accessibilities were also recorded for meals created with whole milk (Figure 3C). Overall, four outliers with fluoride bio-accessibilities above 150% were detected – (a) white bread with peanut butter and jam mixed with both types of milk and (b) white bread with peanut butter mixed with both types of milk. When those were removed, the overall mean (SD) fluoride bio-accessibility of meals when consumed with milk was 71.5% (17.1%). Finally, Figure 3D highlights two outliers – (a) white bread with peanut butter and jam and (b) white bread with peanut butter that resulted in fluoride bio-accessibilities of 164.0% and 129.1%, respectively.

## 4 Discussion

### 4.1 Most commonly consumed food/meals

The first three years of life are generally considered to be the most critical period for dental fluorosis development on permanent maxillary central incisors (5). A comprehensive evaluation of dietary sources was conducted using 3-day food diaries gathered from 91 participants of the IFS at six key time points: 1.5, 3, 6, 12, 24, and 36 months of age. The relatively limited number of diaries was a result of incomplete digitization of these documents and decreasing response rates with increasing age (59). The final list of the most commonly consumed food/meals encompassed both infant and adult dietary sources. In infancy, these sources were almost exclusively focused on breast milk and infant formula expanding to include fruit-based, vegetable-based, and meat-based baby foods, which gradually transitioned towards regular adult foods from approximately 6 months of age. Such results mirrored previous findings on the topic, which identified baby foods, infant formulas, and breastmilk as the main energy sources of children contributing over 99% and 73% to the total energy intake of children between birth and 6 months of age and of children between 6 and 12 months of age, respectively (60). Although commonly consumed (59), breastmilk was excluded from the present study as it poses a low risk for dental fluorosis development due to a very low fluoride content (61–63). Furthermore, children between 6 and 12 months of age are rarely exclusively breastfed (58), favoring intake of infant formulas (64). Therefore, powdered and ready-to-feed (RTF) infant formula alongside various baby foods were used as the main representatives of infancy in this study. In earlier studies, bread, cereals, potatoes, fruit, peanut butter, sweetened beverages, unflavored whole and semi-skimmed milk, along with sweet/salty snacks were among the most commonly consumed energy sources of children up to 4 years of age (65–68), while starches, grains, cereals, and water were identified as the main fluoride sources in early childhood (59). Although based on 3-day food diaries collected in the Iowa Fluoride Study in the 1990s, the findings on the most common dietary sources align with more recent studies, highlighting consistent consumption patterns over time (60, 65–68). Therefore, the assembled list of dietary sources (Supplementary Tables S1, S2) encompassed the main representatives of each food category consumed regularly by young children and provided a sound basis for further bio-accessibility measurements. To calculate fluoride bio-accessibility, an experimental design encompassing three laboratory procedures had to be employed: (a) measurement of fluoride concentration in raw material before *in vitro* digestion; (b) *in vitro* digestion of dietary sources; and (c) measurement of fluoride concentration in supernatants created by *in vitro* digestion.

### 4.2 Fluoride concentration of food/meal items

#### 4.2.1 Individual food items

The mean (SD) fluoride concentration of individually measured food items was 0.518 (1.811) µg/g. Fluoride concentrations of individual food samples analyzed in this study and consumed during infancy are consistent with results found in the literature. For example, a study conducted in Canada in 1982 (69) revealed the fluoride concentration of vegetable-based baby foods between 0.02 and 1.29 µg/g, fruit-based baby foods 0.04–0.39 µg/g, meat-based baby foods 0.05–3.93 µg/g, and cereals 1.24–4.98 µg/g. Heilman et al. (70) reported the fluoride concentration of vegetable-based baby foods to be between 0.01 and 0.42 µg/g, fruit-based baby foods and deserts 0.01–0.49 µg/g, meat-based baby foods 0.01–8.38 µg/g, and cereals 0.01–0.31 µg/g. As for food samples commonly consumed during early childhood, the highest value of 10.730 µg/g was recorded for canned fish in tomato sauce, which aligns with the value recorded for a similar sample in the UK fluoride database (71). The fluoride concentration of fluoridated tap water from Newcastle upon Tyne (0.98 µg/g) matches fluoride levels set by the water fluoridation scheme that aims to achieve 1 mg F/L of water (72). Although in line with previous findings, minor differences in fluoride concentration between samples analyzed in this study and those presented in the literature can be expected. Brand, production site, or the fluoride concentration of the water used for production are just some factors that can impact the final fluoride content (73–76), which highlights the complexity of fluoride measurements. Finally, food samples available in the UK and US markets were analyzed in this study indicating small but detectable differences in fluoride concentration between the different countries (Table 1). Although such variations among different geographical areas are common (73, 77), they should not be ignored. The global movement of goods, including dietary sources, complicates monitoring systemic fluoride exposure and efforts to link total fluoride exposure to health outcomes. This highlights the need for a universal policy on fluoride labelling of food and drink products, especially those consumed in infancy and early childhood (74).

#### 4.2.2 Meal items

Semi-skimmed and whole milk samples were analyzed for fluoride concentration using an indirect method of fluoride determination and a combination of microwave-assisted acid digestion with a direct method of fluoride determination (54). A noticeable difference in fluoride concentration was observed between the two methods. For both whole and semi-skimmed milk, the indirect method of fluoride measurement yielded 0.001 µg/g, whereas the microwave-assisted acid digestion followed by direct method of fluoride measurement resulted in concentrations of 0.029 and 0.047 µg/g for both whole and semi-skimmed milk, respectively. An earlier study found the average fluoride concentration of different samples of homogenized milk to be 0.011–0.035 µg/g, with the highest recorded value being 0.063 µg/g (excluding plant-based milk) (78). Similarly, the fluoride concentration of milk available in Brazil ranged from 0.02–0.07 µg/ml for whole milk and 0.02–0.05 µg/ml for skimmed milk samples (79). Agreement between results obtained using a combination of microwave-assisted acid digestion and those found in the literature prompted usage of specifically these values for further bio-accessibility calculations.

The overall mean (SD) laboratory-measured fluoride concentration of meals was 0.140 (0.213) µg/g, ranging from 0.004 to 1.219 µg/g. Such results are comparable to those obtained by Buzalaf et al. (80) and Pagliari Tiano et al. (81), where the fluoride concentration of meals ranged from 0.007 to 0.580 mg and 0.011 to 0.743 µg/g, respectively. Similarly, the expected fluoride concentration of meals averaged 0.152 (0.219) µg/g, ranging between 0.003 and 0.861 µg/g. While for approximately 50% of samples the expected fluoride concentration of meals was higher than the laboratory-obtained values, suggesting the formation of insoluble complexes between fluoride and other cations potentially lowering fluoride detectability (82), the mean (SD) difference between the two groups was low: 0.012 (0.101) µg/g. A strong positive relationship between laboratory-obtained and expected values was detected through correlation and regression analysis, while a Bland-Altman analysis confirmed the close agreement between the two methods (58) suggesting that they are both valid, produce consistent results, and can be used interchangeably in the future.

Finally, upon examining the groups, the highest laboratory-measured mean (SD) fluoride concentration of 0.442 (0.360) µg/g was observed for meals created with water. This closely matched the expected fluoride concentration of 0.439 (0.379) µg/g. The fluoride concentration of the water used for food preparation is known to affect the overall fluoride concentration of the prepared product. For instance, the UK fluoride database showed varying fluoride levels in food and drink samples prepared with waters of different fluoride concentrations (75). Similarly, Heilman et al. (70) noted variability in the fluoride concentration of analyzed infant foods, identifying the fluoride concentration of the water used for processing as the primary factor, a finding corroborated by Wiatrowski et al. (83).

### 4.3 Fluoride bio-accessibility

#### 4.3.1 NaF standards

Earlier studies on fluoride bioavailability showed complete fluoride bioavailability when NaF was consumed in a fasting state (31) but reduced bioavailability when it was consumed alongside other food items (32, 33, 84). Although the present study also showed 100% fluoride bio-accessibility for pure NaF of the highest concentration (10 µg/ml), a gradual reduction in fluoride bio-accessibility was recorded from 10 to 0.1 µg/ml NaF standards analyzed in this study. Such results are most likely a consequence of a lower proportion of fluoride ions available at lower concentrations when the effect of other, possibly present, cations could have been more significant. The gastric phase of digestion resulted in slightly higher fluoride bio-accessibility when compared to those values obtained after the whole gastrointestinal process. Similar results were recorded in an earlier study when digestion of seafood samples resulted in a reduction of over 50% between gastric and intestinal phases (57), highlighting the significance of complete gastrointestinal digestion when examining the potential effects of fluoride intake (57).

#### 4.3.2 Individual food items

The overall mean (SD) fluoride bio-accessibility for individual food samples was 44.7% (37.5%) with the highest value of 152.3% recorded for jam. Such a value might be considered an outlier as a consequence of jam's sticky texture and high viscosity that might have limited mixing with gastric fluids and enzymes. A study from 2023 conducted using peanut butter indicated that higher mixing forces are needed for the successful digestion of viscous products, therefore favoring dynamic models (85). On the other hand, a result of 150% might not be impossible. Digestion processes might have released more fluoride ions than acid diffusion used for the initial measurement of fluoride concentration (52). The challenging texture of jam might have restricted the contact between the acid and the food item preventing the effective release of fluoride making it undetectable. As for the rest, a majority of samples commonly consumed during infancy and early childhood resulted in fluoride bio-accessibilities below 100%, which emphasizes the limited availability of consumed fluoride for utilization in the human body.

#### 4.3.3 Meal items

The results of this study indicate significant variability in fluoride bio-accessibility depending on the type of beverage consumed alongside meals. Meals consumed with juice and milk exhibited higher fluoride bio-accessibility, averaging 79% and 102%, respectively, while those consumed with carbonated drinks and tap water showed lower bio-accessibility, with averages of 64.3% and 40.2%, respectively. These differences can be partly attributed to the varying fluoride concentrations in the beverages and their distinct chemical properties, which may influence fluoride release and absorption during digestion. As expected, meals prepared with fluoridated tap water generally showed higher fluoride bio-accessibility compared to those made with non-fluoridated water, highlighting the impact of water fluoridation on overall fluoride intake. Meals consumed with milk demonstrated exceptionally high fluoride bio-accessibility, with some outliers exceeding 150%. After removing these outliers, the mean fluoride bio-accessibility for milk-consumed meals dropped to 71.5%, still higher than most other beverage categories. This suggests that milk, particularly whole milk, may enhance fluoride bio-accessibility, possibly due to its fat content or other constituents that influence fluoride absorption. It is interesting to see that peanut butter and jam were common constituents of removed outliers. As explained above, such anomalies could have been a result of a sticky texture and high viscosity characteristic for those products which interfered with digestion and release of fluoride ions (85).

As with individual food samples, the majority of analyzed meal samples resulted in fluoride bio-accessibilities below 100%. Such an outcome can be attributed to the chemical properties of fluoride. Fluoride ions, owing to their electronegativity, tend to form complexes with various cations (57). This must be considered as samples analyzed during this study were mainly complex food matrices containing a variety of ingredients, while the simulated digestive fluids used throughout this project contained a wide range of cations that could have potentially reacted with fluoride rendering it undetectable. In acidic conditions, the creation of hydrogen fluoride (HF) is favored whereas, in alkaline environments, fluoride exists in a dissociated form. Thus, when in its dissociated form, fluoride readily binds with these cations which could result in reduced bio-accessibility, especially during the intestinal phase. This was already proven to be the case above while inspecting the influence of the digestion phase on overall fluoride bio-accessibility from NaF standards. These results showed that the intestinal phase of digestion decreased fluoride bio-accessibility by more than 40% while a reduction of more than 50% between gastric and intestinal phases was observed in an earlier study (57). These results highlight the importance of the entire gastrointestinal process when evaluating the potential risks or benefits of fluoride ingestion.

Furthermore, it should be noted that the static *in vitro* digestion model used in this study, while commonly applied to assess the bio-accessibility of various nutrients (86, 87), has limitations. Unlike dynamic models, which simulate the gradual movement of food through the digestive system, the static model exposes food samples to different digestive phases without changing conditions like pH or enzyme concentration. For example, the gastric pH in this study was adjusted to 1.5–2.5, but in reality, food intake temporarily raises stomach pH (88), potentially reducing fluoride solubilization. One study found that at pH 6, less than 13% of fluoride was soluble when certain seafood types were digested (57).

Overall, these findings highlight the need for careful interpretation of bio-accessibility data, especially given the constraints of the static *in vitro* digestion model and suggest that further research is needed to refine measurement techniques and better understand the factors influencing fluoride absorption in different dietary contexts.

### 4.4 Limitations and recommendations for future studies

This study revealed fluoride bio-accessibilities below 100% for the majority of analyzed food/meal samples, indicating that part of consumed fluoride is unavailable for utilization in the human body. It also underscores the significant variability in fluoride bio-accessibility across different dietary sources, probably influenced by both the chemical composition of the consumed items and the digestion process itself. The static *in vitro* digestion model used for this study fails to fully capture the dynamics of fluoride release and absorption in the human gastrointestinal tract. Furthermore, the complexity of analyzed food matrices could have prevented the release of fluoride ions at any stage of the experiment, rendering it undetectable. Due to their high viscosity and sticky texture, dietary sources like peanut butter and jam further complicated the digestion process and release of fluoride ions highlighting the need for more sophisticated models and experimental designs to accurately measure fluoride content and its bio-accessibility.

Therefore, future studies utilizing dynamic digestion models that more accurately replicate the dynamic nature of human digestion and identify factors influencing fluoride absorption are needed. This is particularly crucial during early childhood, a period when the risk of dental fluorosis is highest. While the present study focused on dietary patterns and fluoride exposures in the USA, future research should encompass a broader range of dietary patterns and regional fluoride exposures. Investigating the impact of other dietary components, such as calcium and iron, on fluoride bio-accessibility is also essential. Furthermore, it is recommended to include all types of milk (e.g., breast milk, plant-based milk, specialized formula) in future studies to provide a more comprehensive understanding of fluoride bioaccessibility in infants.

## 5 Conclusion

Current dietary recommendations for fluoride intake, such as “adequate intake” (AI) and “tolerable upper intake level” (UL) assume that 100% of consumed fluoride is available for utilization by the body. However, this assumption does not align with the observed fluoride bio-accessibility of below 100% detected for the majority of analyzed food/meal samples, as demonstrated in this study.

As the first research of its kind, this study addresses a critical gap in knowledge and provides a foundation for future studies focused on examining factors that can influence fluoride bio-accessibility across diverse populations and dietary sources. Policymakers and health authorities should recognize the significance of these findings and use them to inform future revisions of dietary recommendations providing more precise dietary advice and promoting optimal fluoride intake for overall health. Aligned with Global Target 2.2 of the WHO Global Health Action Plan, this study provides crucial information for shaping evidence-based policy and advancing public health strategies.

Finally, the variability in fluoride content of dietary sources across different geographical regions and food processing methods highlights the significance of systematic monitoring of fluoride intake and introduction of a universal policy on fluoride labelling of food and beverages, particularly due to increased global movement of goods. These measures would enable better consumer awareness and improved public health guidance.

## Data Availability

The original contributions presented in the study are included in the article/Supplementary Material, further inquiries can be directed to the corresponding author.
