# Supplementary material for: Assessment of fluoride bio-accessibility in early childhood diets
Source: Front Oral Health. 2025 Feb 6;6:1526262. doi: 10.3389/froh.2025.1526262 (PMC11839772; doi:10.3389/froh.2025.1526262)
Supplement: Supplementary file 1 [file Datasheet1.pdf]

Supplementary Material

Supplementary Table 1. Fluoride concentrations (µg/g) of individual food samples

Infancy (1.5 – 6 months of age)	
**Food item**	**Fluoride concentration (µg/g)**
Reconstituted formula powder UK	0.011
Reconstituted formula powder US	0.047
Baby food mixed meat UK	0.223
Baby food fruit UK	0.049
Baby food vegetable UK	0.249
Baby food cereals UK	0.173
Baby food fruit US	0.199
Baby food vegetable US	0.228
Baby food cereals US	0.164
RTF formula	0.012
Early childhood (12 – 36 months of age)	
**Food item**	**Fluoride concentration (µg/g)**
Pizza	0.104
Spaghetti	0.069
Hamburger + French fries (McDonald's)	0.360
Macaroni and cheese	0.071
White bread	0.047
Cereals	0.072
Carbonated beverages	0.040
Juice	0.047
Fruit	0.023
Fish in oil	2.633

Fish in tomato	10.730
Fish, prebreaded	0.125
Hot dog	0.875
Milk, whole	0.029
Milk, semi-skimmed (2% milk)	0.047
Instant oatmeal + distilled water	0.050
Potato mashed + distilled water	0.061
Cookies	0.140
Digestive cookies	0.036
Crackers	0.063
Saltine	0.317
Graham	0.217
Peanut butter	0.029
Jam	0.047
Non-fluoridated tap water (Middlesbrough)	0.083
Fluoridated tap water (Newcastle)	0.983
**OVERALL MEAN ± STANDARD DEVIATION**	**0.518 ± 1.811**

Supplementary Table 2. Laboratory-measured and expected fluoride concentration (μg/g) of meal samples

Group	Meal type	Laboratory measured F concentration of meals (μg/g)	The ratio of individual food samples used to create meals	Expected[1] F concentration of meals (μg/g)	Laboratory-measured group mean ± SD	Expected group mean ± SD
**Meals created with carbonated beverages**	Cookies	0.063	0.11:0.89	0.051	0.075 ± 0.034	0.077 ± 0.062
	Pizza	0.058	0.47:0.53	0.070		
	Graham	0.053	0.11:0.89	0.059		
	Saltine	0.063	0.06:0.94	0.056		
	Digestive	0.063	0.06:0.94	0.040		
	Crackers	0.052	0.06:0.94	0.041		

[1] Expected fluoride concentration of meals was calculated as the sum of the fluoride concentrations of individual food samples used for its preparation.

	Fish, prebreaded	0.090	0.31:0.69	0.066		
	White bread + peanut butter + jam	0.045	0.07:0.93	0.040		
	White bread + peanut butter	0.052	0.07:0.93	0.040		
	Hamburger + French fries (McDonald’s)	0.068	0.33:0.67	0.050		
	Macaroni and cheese	0.133	0.44:0.56	0.181		
	Macaroni and cheese + hot dogs	0.154	0.44:0.56	0.231		
**Meals created with juice**	Cookies	0.064	0.17:0.83	0.063	0.078 ± 0.032	0.094 ± 0.079
	Pizza	0.069	0.49:0.51	0.075		
	Graham	0.096	0.17:0.83	0.076		
	Saltine	0.060	0.1:0.9	0.074		
	Digestive	0.042	0.08:0.92	0.046		

	Crackers	0.056	0.1:0.9	0.049		
	Fish, prebreaded	0.073	0.4:0.6	0.078		
	White bread + peanut butter + jam	0.053	0.12:0.88	0.047		
	White bread + peanut butter	0.057	0.12:0.88	0.047		
	Hamburger + French fries (McDonald's)	0.098	0.45:0.54	0.057		
	Macaroni and cheese	0.128	0.57:0.43	0.226		
	Macaroni and cheese + hot dogs	0.144	0.57:0.43	0.290		
**Meals created with whole milk**	Cookies	0.015	0.13:0.87	0.043	0.029 ± 0.030	0.075 ± 0.075
	Pizza	0.021	0.31:0.69	0.052		
	Graham	0.019	0.13:0.87	0.053		
	Saltine	0.016	0.1:0.9	0.058		
	Digestive	0.007	0.12:0.88	0.030		
	Crackers	0.011	0.14:0.86	0.034		

	Cereals	0.013	0.23:0.78	0.039		
	White bread + peanut butter + jam	0.019	0.29:0.71	0.034		
	White bread + peanut butter	0.021	0.29:0.71	0.033		
	Macaroni and cheese	0.082	0.50:0.50	0.195		
	Macaroni and cheese + hot dogs	0.096	0.50:0.50	0.251		
**Meals created with semi-skimmed milk (2% milk)**	Cookies	0.014	0.13:0.87	0.059	0.041 ± 0.052	0.088 ± 0.073
	Pizza	0.023	0.31:0.69	0.065		
	Graham	0.030	0.13:0.87	0.069		
	Saltine	0.024	0.1:0.9	0.074		
	Digestive	0.004	0.12:0.88	0.046		
	Crackers	0.014	0.14:0.86	0.049		
	Cereals	0.011	0.23:0.78	0.049		
	White bread + peanut butter + jam	0.014	0.29:0.71	0.046		

	White bread + peanut butter	0.030	0.29:0.71	0.043		
	Macaroni and cheese	0.120	0.50:0.50	0.204		
	Macaroni and cheese + hot dogs	0.166	0.50:0.50	0.260		
**Meals created with water**	Formula powder UK + Non-fluoridated tap water	0.092	0.13:0.87	0.074	0.442 ± 0.360	0.439 ± 0.379
	Formula powder UK + Fluoridated tap water	0.550	0.13:0.87	0.857		
	Formula powder US + Non-fluoridated tap water	0.103	0.13:0.87	0.079		
	Formula powder US + Fluoridated tap water	0.598	0.13:0.87	0.861		
	Baby food cereals UK + Formula powder UK + Non-fluoridated tap water	0.249	0.25:0.75	0.075		
	Baby food cereals UK + Formula	0.437	0.25:0.75	0.750		

	powder UK + Fluoridated tap water					
	Baby food cereals US + Formula powder US + Non-fluoridated tap water	0.264	0.25:0.75	0.082		
	Baby food cereals US + Formula powder US + Fluoridated tap water	0.728	0.25:0.75	0.756		
	Instant oatmeal + Non-fluoridated tap water	0.099	0.25:0.75	0.075		
	Instant oatmeal + Fluoridated tap water	0.857	0.25:0.75	0.750		
	Potato mashed + Non-fluoridated tap water	0.109	0.17:0.83	0.080		
	Potato mashed + Fluoridated tap water	1.219	0.17:0.83	0.826		

**Meals without common preparation method**	RTF formula + baby food cereals UK	0.133	0.75:0.25	0.052	0.163 ± 0.098	0.118 ± 0.141
	RTF formula + baby food cereals US	0.214	0.75:0.25	0.050		
	Baby food cereals UK + Formula powder UK	0.110	0.25:0.75	0.052		
	Baby food cereals US + Formula powder US	0.177	0.25:0.75	0.076		
	Baby food cereals UK + Baby food fruit UK	0.187	0.25:0.75	0.080		
	Baby food cereals US + Baby food fruit US	0.219	0.25:0.75	0.190		
	White bread + peanut butter + jelly	0.029	0.74:0.13:0.13	0.045		
	White bread + peanut butter	0.045	0.74:0.26	0.043		
	Macaroni and cheese + hot dogs	0.351	0.78:0.22	0.473		

Supplementary Table 3. Fluoride bio-accessibility (%) of individual food items

Infancy (1.5 – 6 months of age)	
**Food type**	**Fluoride bio-accessibility (%)**
Reconstituted formula powder UK	0.1
Reconstituted formula powder US	32.5
Baby food mixed meat UK	25.5
Baby food fruit UK	18.7
Baby food vegetable UK	21.3
Baby food cereals UK	17.3
Baby food fruit US	46.7
Baby food vegetable US	27.8
Baby food cereals US	26.6
RTF formula	25.2
Early childhood (12 – 36 months of age)	
**Food type**	**Fluoride bio-accessibility (%)**
Pizza	48.3
Spaghetti	56.2
Hamburger + French fries (McDonald's)	111.2
Macaroni and cheese	47.8
White bread	108.3
Cereals	28.9
Carbonated beverages	92.1
Juice	78.3
Fruit	124.7
Fish in oil	4.9
Fish in tomato	1.0
Fish, prebreaded	69.5
Hot dog	9.8
Milk, whole	44.4

Milk, semi-skimmed (2% milk)	38.2
Instant oatmeal + distilled water	44.3
Potato mashed + distilled water	62.3
Cookies	13.0
Digestive cookies	28.0
Crackers	20.1
Saltine	22.3
Graham	6.8
Peanut butter	13.8
Jam	152.3
Non-fluoridated tap water (Middlesbrough)	40.9
Fluoridated tap water (Newcastle)	100.2
**OVERALL MEAN ± STANDARD DEVIATION**	**44.7 ± 37.5**

Supplementary Table 4. Fluoride bio-accessibility (%) of meals

Sample name	Average bio-accessible fraction (%)
MEALS WITHOUT COMMON PREPARATION METHOD	
RTF formula + baby food cereals UK	33.1
RTF formula + baby food cereals US	19.7
Baby food cereals UK + Formula powder UK	6.0
Baby food cereals US + Formula powder US	14.9
Baby food cereals UK + Baby food fruit UK	21.4
Baby food cereals US + Baby food fruit US	40.4
White bread + peanut butter + jam	164.0

White bread + peanut butter	129.1
Macaroni and cheese + hot dogs	28.5
**MEAN ± STANDARD DEVIATION**	**50.8 ± 55.9**
MEALS CREATED WITH JUICE	
Cookies + juice	89.9
Pizza + juice	87.5
Graham + juice	64.5
Saltine + juice	84.6
Digestive cookies + juice	127.5
Crackers + juice	91.6
Fish, prebreaded + juice	65.4
White bread + peanut butter + jam + juice	61.3
White bread + peanut butter + juice	95.8
Hamburger + French fries (McDonald's) + juice	42.1
Macaroni and cheese + juice	70.9
Macaroni and cheese + hot dogs + juice	67.2
**MEAN ± STANDARD DEVIATION**	**79.0 ± 21.9**
MEALS CREATED WITH CARBONATED BEVERAGES	
Cookies + carbonated beverages	83.5
Pizza + carbonated beverages	31.0

Graham + carbonated beverages	101.2
Saltine + carbonated beverages	73.3
Digestive cookies + carbonated beverages	59.4
Crackers + carbonated beverages	92.6
Fish, prebreaded + carbonated beverages	41.8
White bread + peanut butter + jam + carbonated beverages	44.5
White bread + peanut butter + carbonated beverages	60.8
Hamburger + French fries (McDonald's) + carbonated beverages	64.5
Macaroni and cheese + carbonated beverages	58.9
Macaroni and cheese + hot dogs + carbonated beverages	59.5
**MEAN ± STANDARD DEVIATION**	**64.3 ± 20.7**
MEALS CREATED WITH TAP WATER	
Formula powder UK + Non-fluoridated tap water (Middlesbrough)	38.8
Formula powder UK + Fluoridated tap water (Newcastle upon Tyne)	65.0
Formula powder US + Non-fluoridated tap water (Middlesbrough)	51.6
Formula powder US + Fluoridated tap water (Newcastle upon Tyne)	66.1
Baby food cereals UK + Formula powder UK + Non-fluoridated tap water (Middlesbrough)	10.0

Baby food cereals UK + Formula powder UK + Fluoridated tap water (Newcastle upon Tyne)	60.8
Baby food cereals US + Formula powder US + Non-fluoridated tap water (Middlesbrough)	0.4
Baby food cereals US + Formula powder US + Fluoridated tap water (Newcastle upon Tyne)	21.7
Instant oatmeal + Non-fluoridated tap water (Middlesbrough)	46.1
Instant oatmeal + Fluoridated tap water (Newcastle upon Tyne)	34.3
Potato mashed + Non-fluoridated tap water (Middlesbrough)	48.7
Potato mashed + Fluoridated tap water (Newcastle upon Tyne)	39.3
**MEAN ± STANDARD DEVIATION**	**40.2 ± 20.1**
MEALS CREATED WITH MILK	
Cookies + milk whole	57.2
Cookies + milk semi-skimmed (2% milk)	51.2
Pizza + milk whole	92.5
Pizza + milk semi-skimmed (2% milk)	86.5
Graham + milk whole	51.0
Graham + milk semi-skimmed (2% milk)	45.0
Saltine + milk whole	66.5
Saltine + milk semi-skimmed (2% milk)	60.5

Digestive + milk whole	72.1
Digestive + milk semi-skimmed (2% milk)	66.1
Crackers + milk whole	64.3
Cereals + milk semi-skimmed (2% milk)	67.1
Cereals + milk whole	73.0
Crackers + milk semi-skimmed (2% milk)	58.3
White bread + peanut butter + jam + milk whole	318.6
White bread + peanut butter + jam + milk semi-skimmed (2% milk)	312.6
White bread + peanut butter + milk whole	166.2
White bread + peanut butter + milk semi-skimmed (2% milk)	160.3
Macaroni and cheese + milk whole	91.9
Macaroni and cheese + milk semi-skimmed (2% milk)	86.0
Macaroni and cheese + hot dogs + milk whole	101.8
Macaroni and cheese + hot dogs + milk semi-skimmed (2% milk)	95.8
**MEAN ± STANDARD DEVIATION**	**102.0 ± 75.8**
